# Reaction of stock market returns to COVID-19 pandemic and lockdown policy: evidence from Nigerian firms stock returns

**DOI:** 10.1186/s43093-021-00080-x

**Published:** 2021-10-06

**Authors:** Isiaka Akande Raifu, Terver Theophilus Kumeka, Alarudeen Aminu

**Affiliations:** Department of Economics, Faculty of Economics and Management Sciences, University of Ibadan, Ibadan, Oyo State Nigeria

**Keywords:** COVID-19 confirmed cases and deaths, Lockdown policy, Stock market returns, POLS, PVAR, G10, G15, G20

## Abstract

Given the effects COVID-19 pandemic on the financial sectors across the world, this study examined the reaction of stock returns of 201 firms listed in the Nigerian Stock Exchange to the COVID-19 pandemic and lockdown policy. We deployed both Pooled OLS and Panel VAR as estimation methods. Generally, the results from POLS show the stock market returns of the Nigerian firms reacted negatively more to the global COVID-19 confirmed cases and deaths than the domestic COVID-19 confirmed cases and deaths and lockdown policy. The results of the impulse response functions revealed that the effects of COVID-19 confirmed cases and deaths and lockdown policy shocks on stock returns oscillate between negative and positive before the stock market returns converge to the equilibrium in the long run. The FEVD results showed that growth in the COVID-19 confirmed cases, deaths and lockdown policy shocks explained little variations in stock market returns. Given our finding, we advocate for the relaxation of policy of lockdown and the combine use of monetary and fiscal policies to mitigate the negative effect of COVID-19 pandemic on stock market returns in Nigeria.

## Introduction

This study investigates the response of stock market returns to COVID-19 pandemic shocks and lockdown policy using 201 Nigerian firms’ data. It is on record that the outbreak of COVID-19 began in the city of Wuhan, Hubei Province of China, on 31 December 2019. Due to its spread to other countries, the World Health Organization (WHO) declared it a global pandemic on 11 March 2020. Since the WHO's declaration, governments across the world have put several measures in place to contain the spread of the virus. Some of these measures include washing of hands with soap, sanitisation of the hands-on regular basis, wearing of nose mask in public places, banning of all socioeconomic, political and religious gathering, restriction of movement within regions and states, banning of international travel, physical and social distancing and total lockdown of the economy [39]. The policy measures may sound good as they contain the health risks; however, the economic implications of some them have begun to manifest in different countries, both developed and developing countries.

As a result of the COVID-19 pandemic and the lockdown policy measures imposed by the governments, most commodity prices and financial asset prices nosedived significantly across the world. The World Bank report that crude oil prices dwindled by 50% between January and March 2020 with WTI benchmark traded at a negative territory for the first time in the history of oil price movement.^1^ The prices of metal such as copper and zinc dropped by 13% and agricultural commodity prices suffered a slight decline during the global lockdown [35]. World Economic Forum [38] estimated the total loss of the financial sector to be around 12.35% globally between January 2020 and May 2020. The Forum further stated that about 9 trillion US dollar has been lost in the financial sector globally. Hospitality industry has suffered the greatest loss of tourists, export revenue and jobs. The UNDP [34] estimated the potential tourists lost to be between 850 million and 1.1 billion. Also, potential export revenue lost is said to be around $910 million and $1.2 billion and jobs lost is around 100 and 120 million jobs. With the regard to unemployment effect of COVID-19 pandemic, ILO (2020a) stated that both formal and informal sectors unemployment have been on the rise across the world. The overall impacts of the COVID-19 pandemic and the measures put in place to contain its spread are the wave of the economic recession that is likely to engulf the countries across the world this year and the coming years [36], c).

**Fig. 1:**
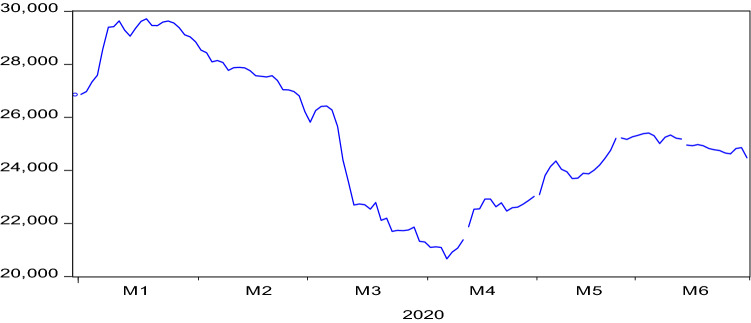
NSE all share index

Nigeria is one of the countries that have been ravaged by the COVID-19 pandemic. The country recorded the first case of the novel COVID-19 on 27 February 2020 [15]. Since then, the country has confirmed a total number 39,539 cases of COVID-19 with the active case revolving around 22,135, about 16,559 patients discharged and 845 deaths also are recorded as at 24 July 2020. This suggests that the recovery and fatality rates are 41.88% and 2.14% in the country. Until recently when the lockdown was relaxed, some states, especially Lagos State-the business hub and the epicentre of the COVID-19 pandemic in the country,^2^ Ogun State^3^ and Abuja-the Federal Capital Territory, were under the total lockdown. During the period of lockdown, the business activities were grounded as there was no interstate movement. Apart from the impacts of the COVID-19 pandemic and lockdown policy on the aggregate economy,^4^ the financial sector experienced some sorts of revenue lost. The stock market capitalisation declined by N13.136 trillion and the investors lost about N 16.88 billion as at June 2020 [3]. All share index (ASI hereafter) as shown in Fig. 1 declined substantially. Specifically, ASI fell by 12.85% from 26,415.54 points in 27 February 2020 to 23,021.01 points on 30 April 2020.

The emergence of COVID-19 pandemic has led to the conduct of some empirical studies on the effect of COVID-19 pandemic shocks on stock market returns [6],Ali, Alam and Rizvi, 2020; [4, 9, 10, 22, 26], Salisu, Ebuh and Usman, 2020; Ahn and Gan, 2020). From these studies, two things are observed. First, most of the studies focused on China and advanced and emerging economies except the study by Ashraf [10] who investigated the response of stock returns to COVID-19 cases and deaths growth in about 64 countries which combined both developed and developing countries in a panel framework. Second, most of the studies used aggregate stock data except the studies by Alfaro et al. [6] and Aravind and Manojkrishnan [9]. Alfaro et al. [6] assess the aggregate and firm’s level stock returns during COVID1-19 pandemic in the USA. Our study is related to these two stands of studies that explored the response of firm’s level stock returns to COVID-19 pandemic in the USA and India [6], the USA [9], India. It, however, differs in terms of scope and implementation procedures. While the study of Aravind and Manojkrishnan [9] focused on the comparison of stock returns before and during the COVID-19 pandemic using descriptive method, the study by Alfaro et al. [6] computed cumulative logistic projection of COVID-19 pandemic and analysed the response of the US aggregate and firm’s stock returns to the projected growth of COVID-19. In this study, we focus on the Nigerian stock market. Specifically, we investigate the effects of COVID-19 confirmed cases and deaths as well as lockdown policy on stock returns of 201 firms listed in the Nigerian Stock Exchange. Most of the studies above focused on advanced economies and some developing and emerging market. In Nigeria, only few studies had assessed the impact of COVID-19 pandemic on stock market returns (see [2], Babarinde, et al. 2020). Albeit, these studies only focused on how COVID-19 cases and deaths affect stock markets without taking into consideration the policy measures taking by government to contain the spread of the virus, the measures, especially the lockdown that have crippled the economic activities affect stock markets. Besides, these studies did not consider the response of stock market returns in Nigeria to global reportage of daily confirmed COVID-19 cases and deaths, thereby neglecting the fact that we are in globalised world where economic activities are linked with one another. Most importantly, aggregate data of the stock market returns are used in most of the studies using static estimation methods except the study by Babarinde [12] that used times series VAR to explore the dynamic effect of COVID-19 confirmed cases and deaths on stock market returns. In our study, we consider both the static and dynamic effects of COVID-19 pandemic on the stock market returns of Nigerian firms. For the static effect, this study uses a Pooled Ordinary Least Squares that accounts for individual firm specific effect and daily specific effect (POLS), fixed effects method (FEM) and random effects method (REM). To trace the dynamic effects, we employ a panel vector autoregressive method (PVAR) estimation method. The PVAR allows us to trace the reaction of stock market returns to the shocks from the COVID-19 confirmed cases and deaths and lockdown policy.

To implement our study, we proceed in three ways. First, we investigate the response of stock market returns of the 201 firms to daily growth in COVID-19 confirmed cases and deaths as reported for Nigeria. As previously mentioned, the country has been recording some COVID-19 cases and deaths. How do daily reported COVID-19 cases and deaths affect the stock market behaviour in Nigeria? It has been established in many advanced and emerging economies that daily news about the number of infected persons and deaths affect the behaviour of the investors which in turn affect negatively the stock market performance [10]. Do the stock market returns respond negatively to daily reported cases and deaths in developing countries such as Nigeria? Moreover, is the effect COVID-19 confirmed cases differed significantly from that confirmed deaths? Second, we also examine how the stock market returns response to the global reported COVID-19 confirmed cases and deaths on daily basis. The financial markets across the world are linked to one another through several channels. Consequently, domestic stock prices usually react to the global events apart from the domestic events. Hence, like any other stock prices from financial sectors across the world, it is possible that the stock prices of the firms listed in Nigerian Stock Exchange might be responding to daily news of cases and deaths related to COVID-19 pandemic in the world. Hence, the need to examine how the stock market returns are responding to daily growth in the number of COVID-19 confirmed cases and deaths in the world. In the case, we used the total world reported COVID-19 confirmed cases and deaths data. Third, lockdown policy aimed at containing the spread of the coronavirus has also affected a lot of economic activities as the movement of the people are restricted. Many firms were shut down and some workers were told to be working from home. Such policy has the potential to affect the trading activities in the floor of Nigeria Stock Exchange and hence affect financial resources of the investors. As a result, we investigate the impact of the policy of lockdown on stock market returns. To capture the lockdown policy, we used the stringency index computed by the University of Oxford in conjunction with the Blavatnix School of Government. The stringency index is made up of COVID-19 prevention measures which include social distancing, cancellation of public events, school closure, workshop closure, public transport closure, stay-at-home orders, public campaigns, restriction of internal movement as well as international travel. The index ranges from 0 and 100 with 0 denoting no lockdown while 100 means a total lockdown [17]. The index has been used in some studies to investigate the impact of lockdown on stock market returns of some groups of countries (see [11]). The main contribution of this study is to compare the response of stock market returns of firms in Nigeria to domestic COVID-19 confirmed cases and deaths with global COVID-19 confirmed cases and deaths as well as policy of lockdown. This is the first study to undertake such studies for Nigeria at firm level.

Following the introductory section, the rest of the study is structured as follows. Section ``Methods'' presents methodology and data sources. Section ``Results and discussion'' discusses the empirical findings while ``Conclusions'' section concludes with policy implications.

## Methods

### Methodology

The broad objective of this study is to primarily investigate the response of stock returns of 201 firms listed in Nigeria stock exchange to the COVI9-19 pandemic and lockdown policy measure. The broad objective is divided into three parts. The first part examines the effects of daily growth in COVID-19 confirmed cases and deaths on the stock returns. This first case of coronavirus infection was reported on 27 February 2020, while the first death due to the COVID-19 occurred on 23 March 2020. The second part examines whether or not the Nigerian firm’s stock returns are affected by the daily growth in the number of COVID-19-related cases and deaths in the world. The third investigates the impact of the lockdown policy measure on stock market returns. The lockdown began on 30 March 2020, in Nigeria. Locking down of the economy led to the restriction of movement of people in the affected areas. The business activities were grounded as the interstate movements were banned, we hypothesise that such lockdown will affect not only human movement but also business activities and in turns affect stock market performance.

The study uses 201 firms listed in the Nigerian Stock Exchange, the organisation that provides a platform for the selling and buying of stocks and financial asset in Nigeria. From the daily closing prices of the firms, we computed daily stock returns using the following formula:

1 $$sr_{{i,t}} = 100*\ln \left( {\frac{{p_{{i,t}} }}{{p_{{i,t - 1}} }}} \right)$$

$sr_{{i,t}}$ is the daily stock returns of a firm $i$ at time $t$. $p_{{i,t}}$ is the current daily closing price of the stock index of a firm $i$ at time $t$, and $p_{{i,t - 1}}$ is the preceding day closing price ($t - 1$) for the firm $i$.

Following Erdem [14] and Ashraf [10], we compute the daily growth rate of COVID-19 confirmed cases and that of lockdown policy as follows:

2 $$cg_{t} = 100*\ln (\frac{{c_{t} }}{{c_{{t - 1}} }})$$

3 $$dg_{t} = 100*\ln (\frac{{d_{t} }}{{d_{{t - 1}} }})$$

4 $$ldp_{t} = 100*\ln (\frac{{ld_{t} }}{{ld_{{t - 1}} }})$$

where $cg_{t}$,$dg_{t}$ and $\text{l} dp_{t}$ are the growths in COVID-19 confirmed cases, deaths and lockdown policy at time $t$, respectively, $c_{t}$,$d_{t}$ and $ld_{t}$ are the number of cases and deaths reported as well as lockdown policy at time $t$ and $c_{{t - 1}}$, $d_{{t - 1}}$ and $ld_{{t - 1}}$ are the cases, deaths and lockdown policy of the preceding day, that is, ($t - 1$). 

We begin the specification of estimation models by specifying POLS model which account for firm specific effect and daily specific effect. Following the Ashraf [10], we specify the POLS as follows:

5 $$sr_{{it}} = \beta _{0} + \beta _{1} \text{cov} _{{cit}} + \beta _{3} ext_{{it}} + \lambda d_{{ft}} + \lambda d_{{dt}} + \varepsilon _{{it}}$$

6 $$sr_{{it}} = \phi _{0} + \phi _{1} \text{cov} _{{dit}} + \phi _{3} ext_{{it}} + \gamma d_{{ft}} + \gamma d_{{dt}} + u_{{it}}$$

7 $$sr_{{it}} = \varphi _{0} + \varphi _{1} ldp_{{pit}} + \varphi _{3} ext_{{it}} + \eta d_{{ft}} + \eta d_{{dt}} + v_{{it}}$$

where $sr_{{it}}$ is the stock market return, $\text{cov} _{{cit}}$, $\text{cov} _{{dit}}$ and $ldp_{{pit}}$ are COVID-19 confirmed cases, COVID-19 confirmed deaths and lockdown policy, respectively. $ext_{{it}}$ is the exchange rate. The exchange rate is used as control variables. It is included in the model because it has a theoretical connection with stock market returns, although the theoretical prediction of expected relationship between exchange rate is yet to be settled [40]. Thus, exchange rate could have positive and negative effect on stock market returns. COVID-19 confirmed cases and deaths as well as lockdown policy are expected to have negative effects on stock market returns. $d_{{ft}}$ and $d_{{dt}}$ are firm specific effect and daily specific effect, respectively. $\varepsilon _{{it}}$,$u_{{it}}$ and $v_{{it}}$ are the error terms that are assumed to be normally distributed with zero mean and constant variance.

We now turn to specify a PVAR model that enables us to trace the response of stock market returns to daily growth of COVID-19 confirmed cases and deaths as well as lockdown policy. The use of panel VAR is important because it is highly helpful when using high-frequency data. The panel VAR also treats the entire variable endogenously and accounts for unobserved individual heterogeneity [23]. Following [1], Love and Zicchino [23], Ouyang and Li [27], we specify the panel VAR as follows:

8 $$\begin{gathered} sr_{{it}} = \Gamma _{0} + \Gamma _{1} sr_{{i,t - 1}} + v_{i} + \varepsilon _{{it}} \hfill \\ i \in \left\{ {1,2,...,N} \right\},\quad t \in \left\{ {1,2,...,T} \right\} \hfill \\ \end{gathered}$$

$sr_{{it}}$ is a vector of three variables, ((cases, deaths or lockdown), stock returns, exchange rate), $\Gamma$ and $\Gamma _{t}$ are the matrix of parameters, $v_{i}$ and $\varepsilon _{{i,t}}$ are the vectors of country-specific panel fixed effects and the error term, respectively. The error term is assumed to be independently and identically distributed with zero mean and constant variance [1]. Using the fixed-effects method to estimate PVAR is usually suffered from the problems of serial correlation and heteroscedasticity. To overcome these problems, we deployed the generalised method of moments (GMM) to estimate the PVAR [23]. To select the maximum lag lengths, we use the MAIC criterion as suggested by Ng and Perron [25]. Thereafter, the orthogonalised impulse response functions (IRFs) is computed applying a Cholesky decomposition method to the residual of the variance–covariance matrix in the PVAR. We also compute the forecast error variance decomposition (FEVD hereafter) which provides information on each of the variables explained by the exogenous shocks to other variables.

### Data sources

We collected daily data from different sources which include ATP Security and Fund Limited, www.ourworldindata.com*,* Government Response Tracker (OXCGRT) and www.investing.com*.* Daily stock prices of the 201 firms were collected from ATP Security and Fund Limited. ATP Security and Fund Limited is a stockbroker company located in Lagos State, Nigeria. It specialises in financial advisory, securities trading and investment management services. As such, it gathers, on daily basis, the stock prices of firms trading on the floor of the Nigerian Stock Exchange. The closing stock prices of the 201 firms were extracted, and the returns for individual firm stock were computed using the formula stated in the methodology section. The daily COVID-19 data on cases of infection and deaths were collected from Our World Data (https://ourworldindata.org/coronavirus).^5^ The Oxford stringency index used to proxy lockdown is obtained from Our World Database. However, the original source of the data is the Oxford COVID-19 Government Response Tracker (see https://www.bsg.ox.ac.uk/research/research-projects/covid-19-government-response-tracker). The data series covers the period from 27 February 2020 to 29 June 2020. The daily exchange rate data, measured in Naira per dollar, were collected from *investing.com.*

## Results and discussion

### Descriptive statistic results

The summary statistics of the series at level is shown in Table 1, while descriptive statistics for growth rate of the variables is presented in Table 2. From Table 1, stock market index has an average value of 48.37, with minimum and maximum values at 0.07 and 10,000, respectively. Under the period of study, average COVID-19 confirmed cases and deaths stood at 11,030 and 265, respectively. The minimum of cases at 1 and minimum deaths at 0, while the maximum values are 41,180 for COVID-19 cases and 868 deaths. On the global level, COVID-19 had an average of over five million cases, with minimum number of 83,381 and maximum number of more than 16 million, while the average deaths are 288,000, with the minimum deaths stood at 2857 and maximum of 600 thousand deaths. The stringency index which measures lockdown has a mean value of 67.3, with a minimum and maximum rigidity index of 11.1 and 85.7, respectively. This suggests that economy was totally lockdown during the ongoing COVID-19 pandemic. Lastly, exchange rate which captures non-stock exogenous shocks has a level average value of 383.8, minimum value around 363.5 and maximum value at about 391.6 under the period of investigation. In terms of skewness, Table 1 shows that exchange rate and stringency are negatively skewed, which means left skewed and left long tail relative to the right tail, whereas the other variables are positively skewed, indicating right long tail relative to the left. Also, stock returns, exchange rate and stringency index exhibit lack of symmetry, while all measures of COVID-19 appear to be symmetry. Considering kurtosis, similar to skewness, Table 1 shows that stock returns, exchange rate and stringency index have heavy-tailed compared to a normal distribution, which signifies the existence of outliers in our data. On the contrary, the other variables have low kurtosis, indicating light tails or lack of outliers.

**Table 1:** Descriptive statistics for level variables

Variables	Obs	Mean	Std.Dev	Min	Max	p1	p99	Skew	Kurt
stockm	21,909	48.373	463.433	0.066	10,000.000	0.111	552.200	14.557	227.155
ncovidtc	21,909	11,030.52	13,002.91	1.000	41,804.000	1.000	41,180.000	0.969	2.555
ncovidtd	21,909	265.174	285.898	0.000	868.000	0.000	860.000	0.728	2.100
wcovidtc	21,909	5,520,000	4,790,000	83,381	1.67e + 07	89,147.000	1.65e + 07	0.694	2.370
wcovidtd	21,909	288,000	209,000	2857.000	660,143.000	3046.000	653,883	0.055	1.706
stigind	21,909	67.319	23.513	11.110	85.650	11.110	85.650	−1.643	4.218
exr	21,909	383.837	8.348	363.47	391.640	364.550	390.57	−1.486	3.556

stockm, ncovidtc, ncovidtd, wcovidtc, wcovidtd, stigind and exr are share price of firms, number of the COVID-19 confirmed cases in Nigeria, number of COVID-19 confirmed deaths in Nigeria, number of the COVID-19 confirmed cases in the World, number of COVID-19 confirmed deaths in the World. stringency index (proxy for lockdown) and exchange rate, respectively

**Table 2:** Descriptive statistics for growth rate of variables

Variables	Obs	Mean	Std.Dev	Min	Max	p1	p99	Skew	Kurt
stockmr	21,708	−0.022	7.237	−110.137	41.622	-26.839	8.168	−8.846	90.567
ncovidtcr	21,708	9.853	17.971	0.000	132.176	0.000	98.083	4.477	26.637
ncovidtdr	18,291	7.435	13.914	0.000	91.629	0.000	91.629	3.937	20.489
wcovidtcr	21,708	4.908	5.245	1.296	33.153	1.382	28.671	2.999	13.924
wcovidtdr	21,708	5.040	6.649	0.676	39.091	0.687	35.123	2.963	13.218
stigindr	21,708	1.678	10.127	−11.546	82.926	−6.127	46.009	6.068	43.508
Exrr	21,708	0.053	0.960	−4.642	4.617	−3.204	4.003	0.177	15.149

stockmr, ncovidtcr, ncovidtdr, wcovidtcr, wcovidtdr, stigindr and exrr are stock market returns of firms, growth rate of number of the COVID-19 confirmed cases in Nigeria, growth rate of number of COVID-19 confirmed deaths in Nigeria, growth rate of number of the COVID-19 confirmed cases in the World, growth rate of number of COVID-19 confirmed deaths in the World. Growth rate of stringency index (proxy for lockdown) and growth rate of exchange rate, respectively

Further, we present summary statistics of our variables in their growth rates and logarithmic returns. The results are displayed in Table 2. As shown in the table, the stock returns have an average value of −0.022 with a standard deviation of 7.2, a minimum return of −110.1% and a maximum return of 41.6%. Exchange rate and stringency index both have average returns of 1.8 and 0.1, with standard deviations of 10.1 and 0.96, respectively. Also, stringency index has minimum and maximum values of −11.5 and 82.9, respectively, whereas exchange rate has a minimum value of −4.61 and maximum value of 4.62. Considering skewness, stock returns exhibit left tailed long skewness relative to the right tail. All the other series are rightly skewed, and long relative to the left tail, except exchange rate, is normally distributed. Further, all the series in their return forms are heavy-tailed, signifying outliers.

### Correlation analysis results

Next is to deal with the problem of collinearity and multicollinearity in the estimation, we examined the correlation matrix among the variables. Table 3 presents the correlation matrix results. Generally, there is negative relationship among the variables except the correlation between COVID-19 confirmed deaths in Nigeria and stock market return in which the correlation is positive and statistically significant. The correlations are all significant at the 5 per cent level. It can be seen from the table that there is no problem of multicollinearity because the values of the correlation among the regressors are relatively moderate.

**Table 3:** Correlation matrices results

Pairwise correlations							
Variables	stockmr	ncovidtcr	ncovidtdr	wcovidtdr	wcovidtdr	stigindr	Exrr
Stockmr	1.000						
Ncovidtcr	-0.578*	1.000					
*p*-value	0.000						
Ncovidtdr	0.220*	0.660*	1.000				
*p*-value	0.000	0.000					
Wcovidtcr	-0.430*	0.614*	0.479*	1.000			
*p*-value	0.000	0.000	0.000				
Wcovidtdr	-0.397*	0.605*	0.509*	0.976*	1.000		
*p*-value	0.000	0.000	0.000	0.000			
Stigindr	-0.298*	0.415*	-0.077*	0.477*	0.466*	1.000	
*p*-value	0.000	0.000	0.000	0.000	0.000		
Exrr	-0.178*	0.160*	-0.073*	0.192*	0.145*	0.113*	1.000
*p*-value	0.000	0.000	0.000	0.000	0.000	0.000	

^*^ shows significance at the 0.05 level

Stockmr, ncovidtcr, ncovidtdr, wcovidtcr, wcovidtdr, stigindr and exrr are stock market returns of firms, growth rate of number of the COVID-19 confirmed cases in Nigeria, growth rate of number of COVID-19 confirmed deaths in Nigeria, growth rate of number of the COVID-19 confirmed cases in the World, growth rate of number of COVID-19 confirmed deaths in the World. Growth rate of stringency index (proxy for lockdown) and growth rate of exchange rate, respectively

### Unit root tests

Further, we carried out three different unit root tests for robustness, that is, the Maddala-Wu panel unit root [24], the Pesaran cross-sectional dependence (CD) [28] and the cross-sectionally augmented IPS (CIPS) [29] tests to evaluate the existence of cross-section dependence and the order of integration of our series. The results of the three tests are presented in Table 4

**Table 4:** Cross-sectional dependence test and cross-sectionally augmented IPS unit root test. *Source* Computed by the Authors

Variable	Cross-sectional dependence (CD)			MW (Chi-sq)		CIPS (Zt-bar)	
	CD test	corr	abs(corr)	Without trend	With trend	Without trend	With trend
stockmr		.		9170.985***	7942.563***	−67.028***	−67.001***
exrr	1486.940***	1.000	1.000	1.8e + 04***	1.6e + 04***	69.312	69.350
ncovidtcr	1486.940***	1.000	1.000	4015.534***	4093.193***	69.312	69.350
ncovidtdr	1486.940***	1.000	1.000	8413.904***	7682.398***	69.312	69.350
wconvidtcr	1486.940***	1.000	1.000	3183.429***	4632.313***	69.312	69.350
wcovidtdr	1486.940***	1.000	1.000	2341.576***	3934.011***	69.312	69.350
stingindr	1486.940***	1.000	1.000	325.768	213.212	69.312	69.350
Dstockmr		.		2.1e + 04***	1.8e + 04***	−67.243***	−67.280***
Dexrr	1480.170***	1.000	1.000	2.5e + 04***	2.0e + 04***	69.312	69.350
Dcovidtcr	1480.170***	1.000	1.000	2.0e + 04***	1.7e + 04***	69.312	69.350
Dcovidtdr	1480.170***	1.000	1.000	2.1e + 04***	1.8e + 04***	69.312	69.350
Dwcovidtcr	1480.170***	1.000	1.000	2.0e + 04***	1.7e + 04***	69.312	69.350
Dwcovidtdr	1480.170***	1.000	1.000	2.1e + 04***	1.8e + 04***	69.312	69.350

stockmr and exrr represent stock returns and exchange rate returns. covidtcr, and covidtdr stand for growth in domestic COVID-19 cases and deaths. wcovidtcr, wcovidtdr and stinginr represent growth in global COVID-19 cases, deaths and lockdown, respectively. D is the first differences of stock returns, exchange rate returns, growth in both domestic and global COVID-19 cases and deaths. MW and CD tests are the first generation tests with Ho of the cross-sectional independence, whereas the second-generation unit root test—CIPS was carried out with the Ho in which the series are I(1). ***signifies1% level of statistical significance. ***, **, and * denote 1%. 5% and 10% level of significant, respectively

It is evident that cross-sectional dependence exists in all the variables when we considered either the logarithm returns for the stock prices and exchange rate or their first differences, as shown by the Pesaran CD test [28], meaning that there exists a correlation between our variables across firms. Further, we test for cross-sectional dependence following Abrigo and Love [1] and Love and Zicchino [23]. We also performed the second-generation unit root test—the CIP test by Pesaran [29], following the confirmation of cross-sectional dependence in all the series. This became necessary given the fact that the first-generation unit root tests^6^ are not consistent in the presence of such an event [13, 32].

As indicated in Table 4, our results show that the order of integration is between I(0) and I(1) with or without trend for all series.

### Panel ordinary least squares results

The results of POLS are presented in Table 5. Although we also have the results of FEM and REM estimations, the results are put in appendix in Table A1. The results consist of five models, model 1 to model 5. Models 1 and 2 capture effects of daily growth in COVID-19 confirmed cases and deaths in Nigeria on stock market returns, respectively. Models 3 and 4 focus on the impacts of daily growth in global COVID-19 confirmed cases and deaths on Nigerian stock market returns while model 5 deals with response of stock market returns to lockdown policy. It can be seen in the model 1 that daily growth in domestic COVID-19 reported cases has a negative and significant effect on stock market returns. Model 2 shows that growth in COVID-19 confirmed deaths has an insignificant positive effect on stock market. The results suggest that generally there is a financial loss during the ongoing COVID-19 pandemic.

**Table 5:** Pooled OLS regression results

	**(Model 1)**	**(Model 2)**	**(Model 3)**	**(Model 4)**	**(Model 5)**
	**Stockmr**	**stockmr**	**stockmr**	**stockmr**	**stockmr**
Ncovidtcr	-0.578***				
	(0.002)				
Ncovidtdr		0.000			
		(0.001)			
wcovidtcr			-3.428***		
			(0.012)		
wcovidtdr				-1.668***	
				(0.008)	
stigindr					-0.024***
					(0.003)
exrr	0.121***	0.000	1.331***	-3.939***	0.000
	(0.030)	(0.017)	(0.031)	(0.041)	(0.030)
constant	7.968***	2.042***	42.617***	5.306***	1.895***
	(0.303)	(0.169)	(0.361)	(0.320)	(0.297)
Obs	21,708	18,291	21,708	21,708	21,708
R-squared	0.858	0.335	0.858	0.858	0.858
firm dummies	YES	YES	YES	YES	YES
daily dummies	YES	YES	YES	YES	YES

Standard errors are in parenthesis; *p < 0.1, **p < 0.05, ***p < 0.01

Similar to the results of model 1, model 3 shows that the growth in global COVID-19 reported cases enters the model negatively and statistically significant at 1% level. However, contrary to the results of model 2, model 4 which examines the effect of daily growth in global COVID-19 reported deaths on stock returns indicates the global COVID-19 confirmed deaths also have a negative and significant impact on stock market returns in Nigeria. This literarily implies that stock market returns react strongly to news of number of infected persons that died globally than the number of persons that died locally. However, a cursory look at the estimated coefficients reveals that the coefficients of cases are greater in models 1 to 4. This suggests that stock market reacted strongly to news of number of persons who contacted corona virus than the number of persons who died of it. Our finding is in line with anticipated or a priori expectations. Given the fact that COVID-19 confirmed deaths come as a result of the virus infection, it follows that stock market participants must have reacted to the confirmed cases earlier than the documented deaths. Similar results are reported by various scholars, such as Abu et al. [2], Ashraf [10], Al-Awadhi et al. [4], Ru et al. [30], Alber [5], among others, who documented that returns on stock markets appear to be more susceptible to confirmed COVID-19 cases than reported deaths. Our results, however, are contrary to findings in Babarinde [12], who document that all the measures of COVID-19 confirmed cases and deaths do not statistically and significantly explain stock prices in Nigeria.

With regard to the results of impact of lockdown policy on stock market returns, we found that lockdown policy negatively and significantly impacts stock market returns with estimated coefficient of impact standing at 0.024%. This is not a surprising result given the fact that promulgation of lockdown policy leads to restriction of movement of the persons and closing down of all economic activities except essential services–agricultural services, medical (doctor) services and security services. This is in line with the submission of Ashraf [11] who hypothesised that the impact of lock down would lead to a negative effect on stock market returns. The reason for this assertion according to him rests on the fact that lockdown and travel restriction aimed at ensuring social distancing has a direct adverse effect on economic activities because places of work such as offices, schools and factories have been shut down. When all these costs are factored in by investors, it is likely to affect their investment negatively.

Concerning the effect of exchange rate on stock market returns, it was found that exchange rate has a positive effect on stock market returns in most of the models. However, significant positive effect only occurred in models 1 and 3. Conversely, in model 4 exchange rate has a negative and significant effect on stock market returns.

### Panel vector autoregression (PVAR) results

**Fig. 2:**
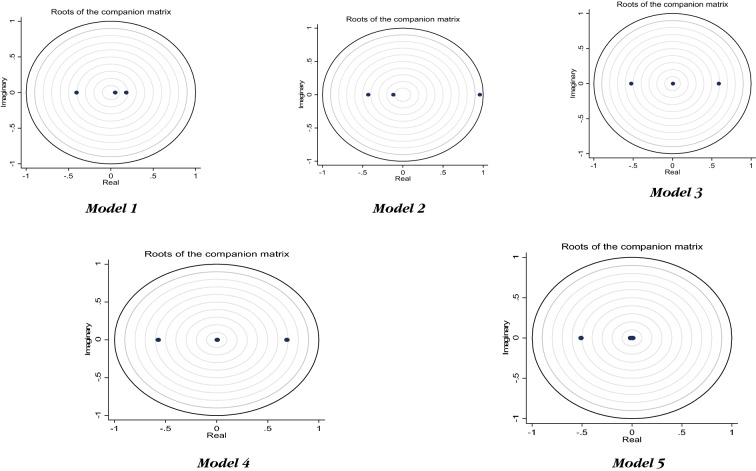
Stability test results

In this section, we presented the results of PVAR and those of its associates such as IRFs and FEVD. However, before presenting the PVAR results, it is expedient to present two preliminary results, namely lag length selection and stability test results. Table 6 presents the results of the lag length selection criteria for PVAR estimation. Regarding the number of an optimal lag length to be selected, we based our choice on the MAIC criterion as suggested by Ng and Perron [25]. As observed from Table 6, three maximum lag lengths were selected. Another test necessary to be conducted before estimating PVAR is stability test. For all our five models, the stability test results are presented in Fig. 2 which consists of 5 graphs each representing models 1 to 5. As shown by the figure, all the models satisfy the stability conditions because all eigenvalues lie within the unit circles.

**Table 6:** Lag order selection criteria

Lag	CD	J	J *p*-value	MBIC	MAIC	MQIC
1	0.9284	2026.934	0.0000	1802.86	1972.934	1912.662
2	0.9814	1968.718	0.0000	1819.335	1932.718	1892.537
3	0.9948	1595.188	0.0000	1520.496	1577.188	1557.097
4	0.9876	–	–	–	–	–

Using the pvarsoc, we generated the following – coefficient of determination (CD), the Hansen’s J statistic (J), with the corresponding probability value (J-P value) [19], the Bayesian information criterion (MBIC), the Akaike information criterion (MAIC), and Andrews and Lu [8]’s Quinn information criterion (MQIC)

Table 7 presents the PVAR results. However, the results are not explained here for our interest is in the responses of stock market returns to innovative shocks from the daily growth in COVID-19 confirmed cases and deaths as well as lockdown policy. Thus, we move to IRFs results and discuss it extensively.

**Table 7:** Panel VAR results

	(Model 1)	(Model 2)	(Model 3)	(Model 4)	(Model 5)
	ncovidtcr	ncovidtdr	wcovidtcr	wcovidtdr	stigindr
*Stock returns*					
lagged stock returns	0.059***	0.955***	0.025***	0.030***	−0.006**
	(0.005)	(0.005)	(0.004)	(0.003)	(0.003)
Lagged COVID-19/stingindr	0.030***	0.001***	0.113***	0.092***	−0.003
	(0.002)	(0.000)	(0.004)	(0.003)	(0.002)
Lagged exchange rate	−0.157***	−0.019***	−0.194***	−0.155***	−0.250***
	(0.018)	(0.004)	(0.009)	(0.008)	(0.007)
*COVID-19*					
Lagged stock returns	−0.008	3.045***	0.076***	0.172***	−0.062***
	(0.015)	(0.033)	(0.003)	(0.004)	(0.004)
Lagged COVID-19/stingindr	0.161***	−0.085***	0.549***	0.646***	−0.009***
	(0.009)	(0.002)	(0.004)	(0.004)	(0.001)
Lagged exchange rate	−1.106***	2.027***	−1.192***	−1.064***	−0.952***
	(0.094)	(0.024)	(0.019)	(0.018)	(0.010)
*Exchange rate*					
Lagged stock returns	−0.034***	−0.015***	−0.032***	−0.035***	−0.023***
	(0.003)	(0.001)	(0.001)	(0.001)	(0.000)
Lagged COVID-19/stingindr	−0.002***	−0.005***	−0.016***	−0.015***	0.002***
	(0.001)	(0.000)	(0.001)	(0.001)	(0.001)
Lagged exchange rate	−0.394***	−0.461***	−0.503***	−0.555***	−0.502***
	(0.010)	(0.002)	(0.007)	(0.006)	(0.002)
Obs	17,085	14,271	17,085	17,085	17,085

Standard errors are in parenthesis. * p < 0.1. ** p < 0.05, *** p < *0.01*

#### Impulse response functions results

The results of the IRFs for the five models are presented and discussed in this section. Specifically, our aim is to analyse the impact of COVID-19 pandemic (domestic and global reported cases and deaths) and lockdown policy on stock market returns in Nigeria. Note that the choice of the proper order of series is the first essential step when carrying out the IRFs estimations. Abrigo and Love [1] suggested that the series coming first in the order of arrangement are more independent and have contemporaneous or a lag effect on the subsequent variables, while the succeeding variables in the model are more dependent affecting the earlier variables with a lag. Following this suggestion, we specify the order of variables where stock returns come last in all models. The IRFs are estimated using Cholesky decomposition of the matrix of the residuals of variance–covariance with orthogonalised shocks as recommended by Holtz–Eakin, Newey and Rosen [20].

Models 1 to 5 present the orthogonalised IRFs of stock prices returns with a 95 per cent confidence interval. In this study, we employed 500 Monte Carlo simulations Gaussian approximation to conduct the IRFs estimations. As stated earlier, we concentrate on the response of stock market returns to shock or innovation in domestic growth of COVID-19 cases and deaths in Nigeria, growth in global cases and deaths from COVID-19 and lockdown policy. As a control variable, we also included the response of the stock market response to shocks in the exchange rate. The IRFs results show the reaction of one variable to innovation or shock in another variable. In addition, the IRFs have the capability of exhibiting the period needed for a variable to converge to its steady state following a shock or innovation.

From model 1, it is observed that following a shock in any of the COVID-19 indicators, there seems to be a nonlinear response from the stock market returns. Specifically, we found that a one standard deviation shock in the growth rate of total COVID-19 cases in Nigeria has a contemporaneous positive effect on the stock returns from the first period to the second period. Stock returns, however, decreased from period 2 to 4 and finally return to equilibrium in the 5th period. In model 2, our results show that shocks to growth in domestic COVID-19 deaths have a positive impact on stock returns in Nigeria. This impact continues from the 1st period to the 10th period without converging to equilibrium. Further, when we consider the impact of a shock to the growth in global COVID-19 confirmed cases and deaths in models 3 and 4, we found that from model 3, our results exhibited similar trends with the domestic cases in Nigeria. Particularly, shocks to global COVID-19 cases have a contemporaneous positive impact on stock returns in the first period. Also, stock market returns responded continuously and positively from period two to period six, but on a diminishing rate, and thereafter converge to it steady state around period seven. Model 4 shows that a shock to the growth in global COVID-19-related deaths also has a simultaneous effect on stock returns, and continues on that trend but on a decreasing rate till around period eight where it converges to equilibrium. Finally, model 5 presents the response of stock returns to lockdown policy. It can be seen that lockdown, which is proxied by Oxford stringency index, has immediate negative effect on stock returns, but the impact diminishes gradually and fizzled out around period three.

**Fig. 3:**
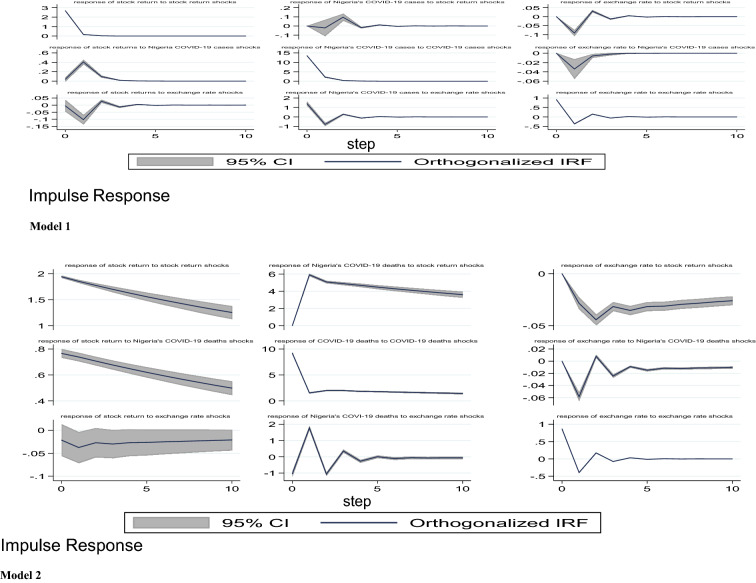

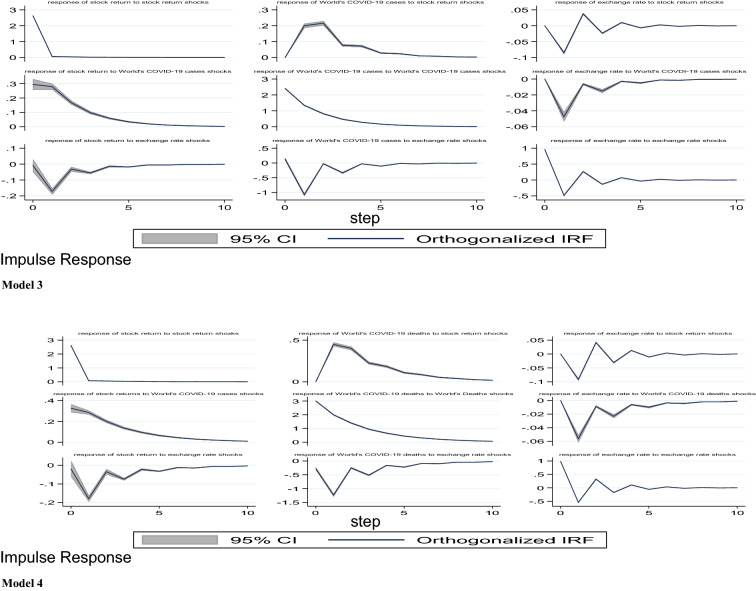

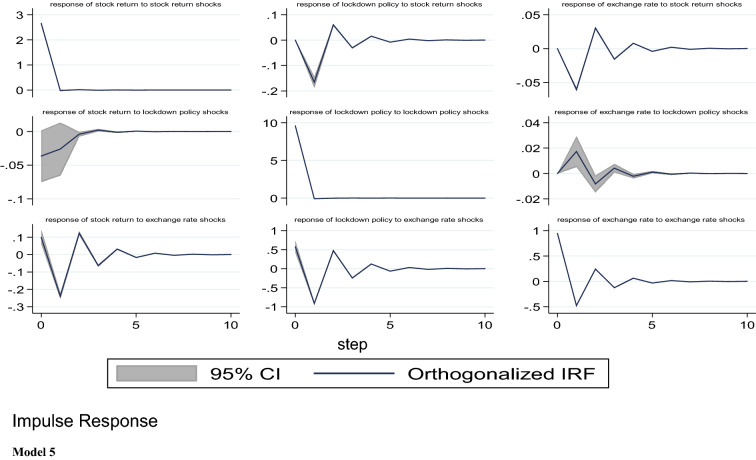
Impulse response functions

From models 1 to 5, the effect of exchange rate on stock market returns is similar. For instance, shocks to exchange causes a negative response from stock returns in the 1st period and this trend continues from period two to period ten. This negative impact from exchange rate in this model appears not to fizzle out after 10th period (Fig. 3).

#### Forecast error variance decomposition analysis

While the IRFs provide relevant information about the impact of deviations in one indicator on other indicators, their major drawback is that they fail to stipulate the scale and quantity of the impact. Consequently, the FEVD estimation was conducted to overcome this drawback. The major advantage the FEVD has over IRFs is that it produces information on the deviation in percentages in the endogenous variables that are designated to both the variable own shock and cross shocks from other variables. It further provides the duration required for a variable to return to its steady state and the role played in attaining that purpose by each variable. Similar to the procedure in PVAR estimations, we conducted the FEVD analysis in five models.

Table 8 presents the results of the orthogonalised FEVD estimates produced from the impulse response coefficient matrices. In model 1, FEVD outputs indicate that stock market returns are explained mainly by its shocks, that is approximately 99.98% in period one, 97.6% in the second period, while moderately reduced in the fifth and tenth periods at approximately 97.5 per cent, respectively. Moreover, we also notice that the growth in total COVID-19 confirmed cases explained about 2.3 per cent of the shocks in stock market returns in period 5 and the tenth period.

**Table 8:** Forecast error variance decomposition results

Response variable	Forecast horizon	Impulse variable		
		Exchange rate	Total cases	Stock market returns
*Model one*				
Exchange rate	1	1.0000	0.0000	0.0000
	2	0.9904	0.0012	0.0084
	5	0.9895	0.0012	0.0093
	10	0.9895	0.0012	0.0093
Total cases (Nigeria)	1	0.0108	0.9892	0.0000
	2	0.0138	0.9862	2.47e-06
	5	0.0142	0.9857	0.0001
	10	0.0143	0.9857	0.0001
Stock market returns	1	3.48e-06	0.0002	0.9998
	2	0.0014	0.0227	0.9759
	5	0.0016	0.0239	0.9745
	10	0.0016	0.0239	0.9745
*Model two*				
Exchange rate	1	1.0000	0.0000	0.0000
	2	0.9954	0.0037	0.0009
	5	0.9903	0.0044	0.0053
	10	0.9850	0.0051	0.0099
Total Deaths (Nigeria)	1	0.0127	0.9873	0.0000
	2	0.0338	0.6887	0.2775
	5	0.0266	0.4664	0.5070
	10	0.0182	0.3613	0.6205
Stock market returns	1	0.0001	0.1347	0.8652
	2	0.0002	0.1358	0.8640
	5	0.0002	0.1365	0.8633
	10	0.0002	0.1367	0.8631
*Model three*				
Exchange rate	1	1.0000	0.0000	0.0000
	2	0.9919	0.0019	0.0062
	5	0.9906	0.0020	0.0074
	10	0.9906	0.0020	0.0074
Total cases (world)	1	0.0029	0.9971	0.0000
	2	0.1330	0.8625	0.0046
	5	0.1291	0.8610	0.0099
	10	0.1297	0.8604	0.0100
Stock market returns	1	0.00001	0.0125	0.9875
	2	0.0042	0.0233	0.9725
	5	0.0047	0.0289	0.9663
	10	0.0048	0.0292	0.9660
*Model four*				
Exchange rate	1	1.0000	0.0000	0.0000
	2	0.9909	0.0025	0.0066
	5	0.9894	0.0027	0.0079
	10	0.9892	0.0028	0.0080
Total Deaths (World)	1	0.0086	0.9914	0.0000
	2	0.1073	0.8790	0.0137
	5	0.1037	0.8723	0.0240
	10	0.1048	0.8706	0.0246
Stock market returns	1	0.00005	0.0154	0.9845
	2	0.0046	0.0267	0.9687
	5	0.0056	0.0359	0.9585
	10	0.0058	0.0370	0.9573
*Model five*				
Exchange rate	1	1.0000	0.0000	0.0000
	2	0.9965	0.0003	0.0032
	5	0.9956	0.0003	0.0040
	10	0.9956	0.0003	0.0041
Stringency index	1	0.0036	0.9964	0.0002
	2	0.0125	0.9872	0.0003
	5	0.0157	0.9840	0.0003
	10	0.0158	0.9839	0.0003
Stock market returns	1	0.0014	0.0002	0.9984
	2	0.0094	0.0003	0.9903
	5	0.0122	0.0003	0.9876
	10	0.0122	0.0003	0.9875

Computed by the authors

Model 2 analyses the contributions of growth in COVID-19 deaths and exchange rate to stock returns. Stock market explained around 86.5% of its own shocks in the first period, 86 per cent in periods two, five and ten, respectively. We also observed that shocks to the growth in domestic COVID-19-related deaths explained about 13.5 per cent of the variations of stock returns in the first period, 13.6 per cent of the fluctuations of stock returns in period two and 13.7 per cent of the changes in stock market returns at the fifth and tenth period, respectively. These results are consistent with previous studies (such as [33],Liu, Manzoor, Wang, Zhang and Manzoor, 2020 among others) and underpinned the fact that global pandemic (in this case COVID-19) is capable of distorting financial markets.

Furthermore, in the case of the global COVID-19 effect on the stock market in Nigeria in model 3, which captures growth in global COVID-19 cases and exchange rate, our results showed that shocks to the growth in global COVID-19 reported cases explained approximately 1.3 per cent of variations in stock returns in period one, around 2.33 per cent of the fluctuations in period two, 2.89 per cent of changes in stock market returns in the fifth period and then increased approximately to 2.92 per cent in the tenth period. Similarly, in model 4, we further observed that shocks to the growth in global COVID-19-related deaths explained around 1.5 per cent of the changes in stock returns in period one, 2.7 per cent in the second period, 3.6 per cent and 3.7 per cent of the variations at the fifth and tenth days, respectively, of the stock market returns forecast error variance. What these results portend is that shocks to the growth in global COVID-19 cases and deaths have a significant influence on the stock market returns in Nigeria.

As regard the lockdown policy as reported in Table 8, model 5, our results indicate that lockdown policy shocks appear to have negligible explanatory effects on the stock market returns, explaining about approximately 0.02 per cent variations in stock returns in period one, 0.03 per cent of the changes in stock returns in periods two, five and ten, respectively. This indicates that the lockdown period imposed by the federal government in Nigeria had negative effects on stock returns as confirmed in ``Impulse response functions results'' section, but does not contribute in explaining the variations in stock market activities. This is possible given the fact that the Nigerian Stock Exchange (NSE) has experienced certain level of developments and innovations in its operations.

## Conclusions

The COVID-19 pandemics related socio-economic costs have raised apprehension to various governments, policymakers and financial sector actors across the world. In this paper, we employed a combination of POLS (fixed/random effects) and PVAR models to investigate the impact of novel COVID-19 confirmed cases and deaths and lockdown policy on stock market returns in Nigeria. The stock returns of 201 firms listed on Nigerian Stock Exchange are examined. We also conducted a series of preliminary tests such descriptive statistics test, correlation analysis and unit root tests. From these series of test, we discovered that the problem of serial correlation did not arise among the variables. Also, evidence of cross-sectional dependence was confirmed in the series and all variables were stationary at first order of integration.

Our main results from the POLS showed the negative effects of COVID-19 reported cases locally and globally on stock market returns. With regard to the growth in domestic COVID-19-related deaths, we also document a depressing impact on the stock market returns, especially the global COVID-19 confirmed deaths. However, the stock market returns in Nigeria reacted strongly to global COVID-19 confirmed cases and death than it reacts to domestic COVID-19 confirmed cases and deaths. Lockdown policy was also found to have a negative and significant effect on stock market returns, suggesting that lockdown also has depressing effect on stock market returns, a financial loss. The IRFs results reveal the existence of nonlinear relationship between stock market returns and the COVID-19 confirmed cases and deaths as the reaction of stock market returns to the COVID-19 pandemic shocks oscillates between negative and positive and converges to the equilibrium in the long run. The results from FEVD analysis show that growth in the COVID-19 confirmed cases, deaths and lockdown shocks explained little variations is stock returns. The main control variable, exchange rate has mixed impacts on stock market returns. However, its negative effect on stock market returns is more significant compared to the positive effect.

Given our results, we suggest that it is expedient for the policymakers saddled with responsibility of seeing to a sound and healthy financial sector need to put policy in place that would make the financial sector resilience to internal or external shocks such as COVID-19 pandemic. Specifically, since stock market return is also reacting negatively to the lockdown measures put in place, it is important for government to relax the lockdown so that business activities can spring up again which in turn would boost the investors’ optimism or confidence. Aside from that, both monetary and fiscal policies could be explored to mitigate the negative effect of COVID-19 pandemic and the policy of lockdown on stocks and shares of the investors. For the fiscal policy, relief package in form of cash must be channelled to those companies which their stocks are affected so that they bounce back to operation and regain the loss suffered due to COVID-19 pandemic. As regards the monetary policy option, the apex bank, the Central Bank of Nigeria, could lower its monetary policy rate which determines the cost of borrowing in the country so that firms can easily have access to cheap funds to resuscitate their operation. This would boost investors’ confidence and optimism in the financial market.

## Data Availability

The datasets used and/or analysed during the current study are available from the corresponding author on reasonable request.

## Appendix

See Table

**Table 9:** Fixed effects and random effects results

	(Model 1)		(Model 2)		(Model 3)		(Model 4)		(Model 5)	
	(FE)	(RE)	(FE)	(RE)	(FE)	(RE)	(FE)	(RE)	(FE)	(RE)
	stockmr	stockmr	stockmr	Stockmr	stockmr	stockmr	stockmr	stockmr	stockmr	stockmr
Total cases (Nigeria)	−0.227***	−0.227***								
	(0.002)	(0.002)								
Total deaths (Nigeria)			0.028***	0.028***						
			(0.001)	(0.001)						
Total Cases (World)					−0.567***	−0.567***				
					(0.009)	(0.009)				
Total deaths (World)							−0.413***	−0.413***		
							(0.007)	(0.007)		
Stringency index									−0.201***	−0.201***
									(0.005)	(0.005)
Exchange rate	−0.660***	−0.660***	0.063***	0.063***	−0.747***	−0.747***	−0.925***	−0.925***	−1.101***	−1.101***
	(0.042)	(0.042)	(0.013)	(0.013)	(0.047)	(0.047)	(0.047)	(0.047)	(0.049)	(0.049)
_Constant	2.249***	2.249***	0.537***	0.537***	2.799***	2.799***	2.107***	2.107***	0.374***	0.374***
	(0.046)	(0.045)	(0.015)	(0.015)	(0.061)	(0.061)	(0.056)	(0.056)	(0.047)	(0.047)
Obs	21,708	21,708	18,291	18,291	21,708	21,708	21,708	21,708	21,708	21,708
R^2^	0.341	0.341	0.050	0.050	0.194	0.194	0.173	0.173	0.110	0.110
F-stat	5573.25	11,240.84	475.77	953.88	2591.30	5227.25	2242.22	4523.15	1327.94	2678.94
*p*-value	(0.000)	(0.000)	(0.000)	(0.000)	(0.000)	(0.000)	(0.000)	(0.000)	(0.000)	(0.000)
Hausman test	0.000	0.000	0.000	0.000	0.000					

Standard errors are in parenthesis. ** p* < *0.1, ** p* < *0.05, *** p* < *0.01*

^9^.

## Abbreviations

ASI: All share index
CD: Cross-sectional dependence
CIPS: Cross-sectionally augmented IPS
COVID-19: Severe acute respiratory syndrome coronavirus 2 (SARS-CoV-2)
ECDC: European Centre for Disease Prevention and Control
FCT: Federal Capital Territory
FEM: Fixed effects method
FEVD: Forecast error variance decomposition
GMM: Generalised method of moments
ILO: International Labour Organization
IRFs: Impulse response functions
MAIC: Modified Akaike information criteria
MBIC: Modified Bayesian information criteria
MQIC: Modified Quinn information criteria
NSE: Nigerian Stock Exchange
OXCGRT: Oxford COVID-19 Government Response Tracker
POLS: Pooled ordinary least squares
PVAR: Panel vector autoregression
REM: Random effects method
UNDP: United Nations Development Programme
US: United States
VAR: Vector autoregression
WEF: World Economic Forum
WHO: World Health Organization
WTI: West Texas Intermediate

## Footnotes

1 The energy prices cascaded down owing to the drop in demand for crude oil by some key sectors of the economy, most importantly the transport industry which activities have been grounded by restriction of movement locally and internationally.

2 Out of 39,539 causes recorded so far at 24 July 2020, Lagos State had a total of 14, 177 cases, 2,087 recovered and 192 patients died.

3 Ogun State was in total lockdown due to its proximity to Lagos-the epicentre of the COVID-19 pandemic.

4 See Farayibi and Asongu [15] for details of the effects of COVID-19 pandemic on the Nigerian economy.

5 Our World Data also gathers the COVID-19 data from the European Centre for Disease Prevention and Control (ECDC).

6 MW panel unit root test by Maddala& Wu (1999) and Pesaran cross-sectional dependence (CD) [28]
